# Development of the care programme for the last days of life for older patients in acute geriatric hospital wards: a phase 0–1 study according to the Medical Research Council Framework

**DOI:** 10.1186/s12904-015-0025-z

**Published:** 2015-05-09

**Authors:** Rebecca Verhofstede, Tinne Smets, Joachim Cohen, Massimo Costantini, Nele Van Den Noortgate, Agnes van der Heide, Luc Deliens

**Affiliations:** End-of-Life Care Research Group, Vrije Universiteit Brussel (VUB) & Ghent University, Brussels, Belgium; Palliative Care Unit, IRCCS Arcispedale S. Maria Nuova, Reggio Emilia, Italy; Department of Geriatrics, Ghent University Hospital, Ghent, Belgium; Department of Public Health, Erasmus MC, Rotterdam, The Netherlands; Department of Medical Oncology, Ghent University Hospital, Ghent, Belgium

**Keywords:** Liverpool Care Pathway, End-of-life care, Older people, Hospital

## Abstract

**Background:**

The effects of the Liverpool Care Pathway (LCP) have never been investigated in older patients dying in acute geriatric hospital wards and its content and implementation have never been adapted to this specific setting. Moreover, the LCP has recently been phased out in the UK hospitals. For that reason, this study aims to develop a new care programme to improve care in the last days of life for older patients dying in acute geriatric wards.

**Methods:**

We conducted a phase 0–1 study according to the Medical Research Council Framework. Phase 0 consisted of a review of existing LCP programmes from the UK, Italy, and the Netherlands, a literature review to identify key factors for a successful LCP implementation and an analysis of the concerns raised in the UK. In phase 1, we developed a care programme for the last days of life for older patients dying in acute geriatric wards based on the results of phase 0. The care programme was reviewed and refined by two nurses and two physicians working in an acute geriatric ward and by two experts from Italy and the Netherlands.

**Results:**

Phase 0 resulted in the identification of nine important components within the LCP programmes, five key factors for a successful LCP implementation and a summary of the LCP concerns raised in the UK. Based on these findings we developed a new care programme consisting of (1) an adapted LCP document or Care Guide for the older patients dying in an acute geriatric ward, (2) supportive documentation, and (3) an implementation guide to assist health care staff in implementing the care programme on the acute geriatric ward.

**Conclusions:**

Based on the existing LCP programmes and taking into account the key factors for successful LCP implementation as well as the concerns raised in the UK, we developed a care programme for the last days of life and modelled it to the acute geriatric hospital wards after gaining feedback from health professionals caring for older hospitalized patients.

## Background

Ageing [1-4] and the increasing prevalence of chronic and degenerative conditions [5] imply that a growing number of older people in developed countries will need palliative care. The World Health Organization has recently identified palliative care as one of the public health priorities for older people [6].

Despite the fact that the majority of older people prefers to die at home [7] and the increasing importance of the nursing home as a place of end-of-life care and dying [8], a large proportion of the aged population (>70 years) die in a hospital [9] where palliative care goals and principles are often achieved with difficulty [10-12]. Previous studies have shown the poor quality of care delivered to the older population at the end of life, especially in the hospital setting [13, 14].

Several end-of-life care pathways have been developed to improve the quality of end-of-life care [15-17]. The Liverpool Care Pathway (LCP) for the Dying Patient is one such pathway. It was developed in 1997 in the United Kingdom (UK) as a multi-professional document that provides a template of care for the final days and hours of life and aims to transfer the hospice model of care to mainstream hospital services [15, 18]. The LCP is based on the principles of palliative care: regular assessment and management of symptoms, comfort measures, effective communication with patients, and their families, and provision of psychological, social, and spiritual/existential support. It focuses on the individual physical, psychological, and spiritual needs of the dying patient and their family during the last hours and days of life and provides health care professionals with guidance on the different aspects of care required, including comfort measures, anticipatory prescribing of medications, discontinuation of inappropriate interventions, and the psychological and spiritual/existential support of the patient and family [18].

Although studies suggest that the LCP can improve the quality of end-of-life care in a cancer population [19-22], its effectiveness in people dying of causes other than cancer, especially older people, has not yet been investigated. Furthermore, although the LCP as developed in the UK is meant to be implemented in every health care setting, the provision, and organization of end-of-life care can vary between health care settings and specific patient populations should be taken into account [6]. It is for instance known that the recognition of the dying phase is more challenging in older non-cancer patients [15] and that around half of older patients in hospital are cognitively impaired [23]. Older people dying in hospital are thus a specifically vulnerable patient group for which end-of-life care can be significantly improved [13, 14]. Hence, if we want to introduce and use a care programme for the last days of life in acute geriatric hospital wards, the context should also be taken into account, especially during the process of implemention. As the LCP has been widely criticized since June 2012 for failing to help physicians and nurses provide appropriate care, the development of an adapted care programme for the last days of life should also take into account the concerns that have been raised in the UK. Raised concerns regarding the LCP arise mainly from inappropriate implementation and use and not the principles of the LCP itself. This was also recently highlighted in an independent review which recommended phasing out the LCP in the UK by July 2014 [24].

This study aims to develop a new care programme to improve care in the last days of life of older people dying in acute geriatric wards.

## Methods

### Study design

To develop a care programme for the older hospital population to improve care in the last days of life the Medical Research Council (MRC) Framework was used. The MRC Framework is an approach aimed at providing a robust methodological basis to the development and evaluation of complex interventions [25]. According to the MRC Framework, interventions should be developed, and tested systematically using a phased approach [26, 27]. In this study we aimed to complete the first two phases: phase 0 and phase 1.

The study is approved by the Ethics Committee of the University Hospital of Ghent University, the Central Ethics Committee of the University Hospital of the Vrije Universiteit Brussel and by the Local Ethics Committees of the participating hospitals in Flanders.

### Phase 0: preclinical phase

The preclinical phase consisted of a review of existing LCP programmes, a literature review to identify key factors for a successful LCP implementation and an analysis of the concerns regarding the use of the LCP in the UK.

#### Review of existing LCP programmes

We first reviewed the LCP programme developed in the UK, which is a Continuous Quality Improvement Programme to be implemented, disseminated, and sustained according to the Service Improvement Model, moving on 4 phases, and incorporating 10 different steps [18]. The development of our care programme is also based on this theoretical approach. Also the LCP programmes developed in the Netherlands and Italy, which are based on the original LCP programme from the UK, were selected to be reviewed. The aim of the review was to identify the different components of the LCP programmes, to compare them, and to identify useful components for the development of our care programme for older patients dying in acute elderly wards. Specific reasons have guided the selection of these programmes: the LCP programme from the UK was the one originally developed [18, 28], the Dutch LCP programme uses similar language to that of Flanders [29] and the Italian LCP programme [30] is, to our knowledge, the only LCP programme which has been rigorously evaluated using a controlled trial design [20, 21, 30-33].

#### Review of literature to identify key factors affecting a successful LCP implementation

A PubMed literature search on LCP implementation in the hospital setting was conducted. The search used the terms ‘Liverpool Care Pathway’, ‘hospital’, and ‘implementation’. Studies were included if they were published in English, performed in a hospital setting and if they provided an explanation of the process of implementation, such as facilitating factors, or barriers. As the LCP was developed only in the late 1990s we limited our search to relevant literature dated from 1998 to December 2012. The literature retrieved was examined in depth and key factors for a successful implementation of the LCP were identified.

#### Analysis of the concerns regarding the LCP in the UK

Our methodology consisted of a close follow up of the media concerns by all involved researchers. We collected and read related reports about the criticisms of the LCP in the UK disseminated in press coverage or published on PubMed between the onset of the public discussion (October 2012) and the development of our care programme (March 2013) [34-41]. During several meetings we discussed the reports with each other and aimed to deduce the main concerns about the use of the LCP. The raised concerns were subsequently discussed with clinicians from the UK, Italy, and the Netherlands.

### Phase 1: modelling phase

We developed and modelled a care programme for the last days of life for the older hospital population using the different identified components of the LCP programmes reviewed and the key factors for successful LCP implementation and taking into account the concerns raised regarding the LCP in the UK. In order to take into account the specificities of the older hospital population and the setting in which they are cared for, the preliminary programme was reviewed by two nurses caring for older hospitalized patients, two geriatricians, and one internal medicine physician. Also experts from the UK, Italy, and the Netherlands were involved in this phase: clinicians and a psychologist responsible for the coordination of project implementations. Five researchers, consisting of one geriatrician, three sociologists, and one nurse, discussed all the input gathered and the feedback of the reviewers and used the results of this discussion for the refinement of the programme. As it is not embedded in our culture to involve family carers in developing care improvement strategies, there was no public involvement.

## Results

### Phase 0: preclinical phase

#### Review of existing LCP programmes

The review of the original LCP programme developed in the UK [18, 28] and the LCP programmes used in Italy [30] and the Netherlands [29] identified three common documents: 1) an LCP document, 2) supportive documentation, and 3) an implementation guide.

1. The LCP document
  The original LCP document was developed in 1997 in the UK and has regularly been updated in accordance with the latest evidence. The latest LCP generic version 12 was launched in December 2009 and can be used in all health care settings where end-of-life care needs to be provided.
  An algorithm in the LCP document is included to support the clinical decision making process regarding the recognition and diagnosis of dying and the appropriate use of the LCP to support care in what are thought to be the last hours or days of life. The LCP can be used when the multidisciplinary team-physicians, nurses, and allied health professionals treating a patient-has agreed that the patient is dying and all reversible causes for the current situation have been considered. Recognizing and diagnosing the last days and hours of life is complex and a second opinion or the support of a palliative care team may be required. When the LCP is initiated, the focus of care changes to care of the dying, including discussion with the family carer and when possible the patient. The current plans of care need to be reviewed and inappropriate interventions stopped when the burden is greater than the benefits. The LCP includes a regular assessment process. If the patient improves and is deemed not to be dying by the multidisciplinary team, the LCP can be stopped [18].
  The LCP document lists a number of care goals that guide health professionals to focus on the major issues that are likely to be relevant for patients and their families in the last hours or days of life. Each care goal is accompanied by prompts in order to help health care staff to better understand the content and importance of the care goal. The LCP document consists of three sections [18]:
  Section 1: initial assessment. This section is to be completed when the multidisciplinary team estimates that the patient has entered the dying phase. This section deals with anticipatory prescription of important medications, discontinuation of inappropriate interventions, spiritual/religious assessment, and appropriate information-giving and communication with patients, family, and others.
  Section 2: ongoing assessment. This section focuses on regular assessment of important indices of comfort for the dying patient and their family including symptom control and maintaining the ongoing physical, psychological, and spiritual/religious/existential comfort of the patient and family.
  Section 3: care after death. This section focuses on the assessment of important practical issues and appropriate support for family carers after the death of the patient.
  Care goals are to be documented as either ‘achieved’, ‘not achieved’, or, where appropriate ‘not applicable’. Where ‘not achieved’ is documented, the care professional must make notes concerning the cause or reason and detail the course of action taken. “Not achieved” care goals are not seen as negative but highlight the importance of clinical skills in deciding to deviate from the suggested plan of care in response to individual patient needs [18, 42]. An accurate documentation of ‘not achieved’ care goals ensures that each of them can be tracked and monitored [43].
  In Italy, an earlier LCP hospital version 11 was translated in compliance with the original UK format and approved by the LCP Central Team of the Marie Curie Palliative Care Institute Liverpool (MCPCIL) [20]. The content and structure of the Italian document is very similar to the UK LCP document.
  In the Netherlands, the LCP generic version 12 was translated into Dutch and substantive changes were made. In the Dutch LCP document some care goals were considerably modified or deleted [44]. The care goals concerning clinically assisted nutrition and hydration are less prominently presented, the care goal concerning the maintenance of the patient’s skin integrity was deleted and the form of documentation of the care goals was changed to ‘yes’ or ‘no’ instead of ‘achieved’ or ‘not achieved’.
2. Supportive documentation
  In all three countries supportive documentation has been developed. These documents consist of a goal data dictionary and information leaflets for health care professionals and family carers in support of the LCP document. The goal data dictionary, originally developed in the UK, is designed for health care staff to fully understand the care goals stated in the LCP document and to guide them in correctly recording “not achieved” care goals. In all three countries information leaflets were developed. A leaflet for health care professionals about the LCP was developed in the UK and the Netherlands. The following leaflets for family carers were developed: a leaflet about communication, medication, comfort, and reduced need for food and drink to be given following a discussion regarding the plan of care (UK only), a leaflet about the entering of the dying phase (UK and the Netherlands), a leaflet about the facilities in the health care setting (UK, Italy and the Netherlands), a leaflet about grief, and bereavement after the patient’s death (UK, Italy, and the Netherlands), and a leaflet about practical arrangements after the patient’s death (Italy only).
3. Implementation guide
  An implementation guide to assist health care staff in correctly implementing the LCP document and its supportive documentation within a health care setting was developed in all three countries. We identified nine components in the implementation guides used in the UK, Italy, and the Netherlands: 1) establishing the LCP implementation project and preparing the environment for organizational changes, 2) preparing the documentation, 3) baseline review, 4) training health care staff, 5) LCP use, and ongoing support, 6) reflective practice, 7) evaluation, 8) continuing development of competencies and 9) ongoing education, training and support. Consistencies, and differences in the components of the different implementation guides are listed in Table 1.

**Table 1:** Consistencies and differences in the components of three LCP implementation guides

**United kingdom**	**Italy**	**The Netherlands**
**Component 1-Establishing the LCP implementation project and preparing the environment for organizational change**		
1. Informing all relevant clinical staff^1^	1. Informing all relevant clinical staff	1. Informing all relevant clinical staff
2. Executive endorsement	2. Executive endorsement	2. Executive endorsement
3. Involvement of specialist palliative care services is recommended	3. Involvement of specialist palliative care services is obvious: Palliative Care Unit (PCU) is responsible for implementation	3. Involvement of specialist palliative care services is recommended
4. LCP facilitators^2^: members of the ward	4. No LCP facilitators: PCU is responsible for implementation	4. LCP facilitators: members of the ward
5. Steering group^3^: members of the ward	5. Steering group: PCU with two reference persons as a link between ward and PCU	5. Steering group: members of the ward
6. Intensive training^4^: of LCP facilitators	6. Intensive training of PCU	6. Intensive training of LCP facilitators
7. Project registration with LCP Central Team (UK), LCP National Centre (Italy), or Comprehensive Cancer Centre of the Netherlands		
**Component 2-Preparing the documentation**		
Adapting the LCP document and/or supportive LCP documentation to the ward^5^		
**Component 3-Baseline review**		
Analyzing end-of-life care data and feedback the results to the staff^6^		
**Component 4-Training health care staff**		
1. LCP facilitators and specialist palliative care colleagues train health care staff	1. Health care staff follow a mandatory 12 h training organized by PCU	1. LCP facilitators and specialist palliative care colleagues train health care staff
2. Aim training	2. Aim training	2. Aim training
○ To understand and work with LCP	○ To understand and work with LCP	○ To understand and work with LCP
○ document	○ document	○ document
○ An education in LCP related issues^7^	○ An education in LCP related issues	
**Component 5-LCP use and ongoing support**		
1. LCP use after sufficient training and education	1. LCP use after sufficient training and education	1. LCP use after sufficient training and education
2. Ongoing support and supervision of LCP facilitators each time the LCP document is used^8^	2. Intensive support and supervision of PCU through repeated coaching, telephone, and direct guidance, discussion of clinical cases, and clinical audits	2. Ongoing support and supervision of LCP facilitators each time the LCP document is used
**Component 6-Reflective practice**		
1. To engage staff in ongoing and reflective practice^9^	1. Semi-intensive support and supervision of PCU through repeated coaching, telephone, and direct guidance, discussion of clinical cases, and clinical audits	1. To engage staff in ongoing and reflective practice
2. To develop and deliver ongoing and sustainable education strategies		2. To develop and deliver ongoing and sustainable education strategies
**Component 7-Evaluation**		
1. To organize a formal and quantitative reflection (= audit) ^10^	1. To organize a qualitative evaluation of implementation^11^	1. To organize a formal and quantitative reflection (= audit)
2. The audit acknowledges areas where further education or training is needed	2. The qualitative evaluation acknowledges areas where further support, education, or training is needed	2. The audit acknowledges areas where further education or training is needed
**Component 8-Continuing development of competencies**		
To develop knowledge and skills of staff constantly to embed LCP model within the ward^12^	PCU supports ward staff through repeated coaching, telephone, and direct guidance, discussion of clinical cases, and clinical audits	To develop knowledge and skills of staff constantly to embed LCP model within the ward
**Component 9-Ongoing education, training, and support**		
To create structures and processes to underpin the continuing education, training, and support required		
Examples:		
❖ To link with local audit departments to encourage ongoing reflection on the quality of care delivery		
❖ To keep up to date with developments in end of life care		
❖ To encourage ongoing liaison with local specialist palliative care teams		
❖ To participate in regional and national audit		

^1^All clinical staff are to be informed about the project and made aware of the importance to change the care in the last days of life.

^2^LCP facilitators are assigned to preside the steering group.

^3^A steering group needs to be established to coordinate the project and consists of members of the ward who are motivated for this project or the PCU with two reference persons (Italy).

^4^LCP facilitators or PCU (Italy) are intensively trained in order to provide leadership for the project.

^5^The ward implementing the LCP can adapt the LCP document and/or supportive LCP documentation to the local health care setting if these adaptations are approved by the LCP Central Team, LCP National Centre, or Comprehensive Cancer Centre of the Netherlands (i.e. adapting prompts of care goals, adding care goals, adapting information leaflets, local design of information leaflets).

^6^To highlight and reinforce the need for change within the ward, it is important to retrospectively evaluate the care during the last days of life by reviewing the medical and nursing files and giving feedback about these results to the staff.

^7^Training and education is also related to competencies important for good care during the last days of life (i.e. communication, symptom control).

^8^Ongoing support and supervision each time the LCP document is used for a dying patient, is necessary to increase staff’s knowledge and confidence in using the LCP and empower them in caring for the dying.

^9^Reflections on the LCP document use and the specific elements of care delivery provide an opportunity to acknowledge which competencies need to be maintained and which need to be improved.

^10^The first LCP documents are quantitatively evaluated in order to provide feedback, highlight improvements since the implementation and identify areas where further education or training is needed.

^11^The PCU qualitatively evaluates and discusses the performance and progress of each of the previous components in order to identify staff’s training needs and barriers for the LCP use and provision of optimum end-of-life care.

^12^Solutions for identified training needs and barriers are to be sought and performed in order to embed the LCP programme within the organization.

#### Identification of key factors for a successful implementation of the LCP

The PubMed search on key factors affecting a successful LCP implementation in the hospital setting resulted in 15 records. The title and abstract of all these records were screened for inclusion criteria and five full-text articles were retrieved for detailed evaluation from which five key factors for a successful LCP implementation could be identified. These factors are:

1) having a dedicated facilitator to provide training and ongoing support on the hospital ward about the benefits and goals of the LCP [45-47], 2) training and ongoing education on why and how to use the pathway, for nurses, and especially physicians [45-48], 3) the organization of an audit and of feedback opportunities [45, 47], 4) having a central coordinating LCP office to support local LCP facilitators [49] and, 5) funding, and time for efforts such as facilitation, education, and training [45, 46].

#### Analysis of the concerns regarding the LCP in the UK

Since the second half of 2012 family carers of dying patients in the UK had begun to express their concerns regarding the LCP. Reports in some newspapers suggested that thousands of patients were being put on the pathway and were having treatment, including hydration, and nutrition, withheld because they were difficult to manage and in order to free up beds [34].

Family carers also expressed their concerns because the LCP was often used without their consultation or knowledge [35, 36].

Supporters of the LCP reiterated that the LCP is not about ending life but about delivering excellent end-of-life care and published a statement refuting the misconceptions about the pathway [34, 37]. Nevertheless, it was acknowledged that there were some problems with it. Consequently, an independent review of the concerns regarding the LCP was performed to better understand them and to investigate ways in which the LCP has worked well [36]. As that review was only published in July 2013, five months after the initiation of our study, we could not use its findings for the development and modelling of our care programme. Nevertheless, we were able to deduce the main concerns over the use of the LCP from reports, letters, reviews, and views disseminated in the media and published on PubMed. The concerns centered mainly around (1) improper or poor implementation of the LCP leading to cases of inadequate end-of-life care [34, 35, 38], (2) unacceptable and inadequate communication with the patient and/or family carers [35, 39, 40], (3) the LCP being used as a tick box exercise [41], and (4) the use of the term ‘pathway’ which created the perception that a patient has to die once they are placed on the pathway [34, 35, 38].

### Phase 1: modelling phase

Results of the phase 0 were used to develop the care programme for the last days of life for the older hospital population.

It consists of the following parts: (1) a Care Guide for the Last Days of Life, (2) supportive documentation, and (3) an implementation guide.

1. Care Guide for the Last Days of Life
  We first developed a care guide for the older hospital population. The original LCP generic version 12 from the UK was translated into Flemish in compliance with the original format. Afterwards the translation was grammatically compared with the Dutch LCP version and improved in terms of wordings. The translated document was then reviewed for legibility, usability, and applicability by two nurses caring for older hospital patients, two geriatricians, and one internal medicine physician, which led to a number of adaptations in order to refine and improve the document. A first adaptation concerned the change of the name ‘Liverpool Care Pathway’ into ‘Care Guide for the Last Days of Life’. According to the reviewers this change was crucial to avoid misconceptions about the true nature of the LCP, as the term ‘care pathway’ was perceived as a protocol rather than an approach to care. This change directly addressed one of the identified concerns regarding the LCP in the UK. A second refinement was the adaptation of the care goals to the older hospital population and the setting in which they are cared for. Table 2 lists these adaptations for section 1 and 2 of the Care Guide. Thirdly, the reviewers suggested the Care Guide should be shortened as the document was perceived as bulky. Therefore the introductory part of the document (i.e. the information for family carers concerning communication, medication, comfort, and reduced need for food and drink and information for health care professionals about the LCP) and the prompts illustrating the care goals were left out of the care guide. The information for family carers is instead presented in a separate information leaflet for family carers and the prompts illustrating the care goals are placed in a separate goal data dictionary. To improve the readability of the Care Guide, a fourth refinement was made, namely highlighting in different colours the care goals to be interpreted by physicians and those to be interpreted by nurses.

**Table 2:** **The adaptation of the care goals of the UK LCP version 12 to the older hospital population**

**Section**	**Subsection**	**Goal**	**Changes**
1	Communication	1.1	▪ Reworded questioning under this care goal:
			‘Does the patient have an expressed wish for organ/tissue’ replaced by ‘Does the patient have an expressed wish to donate his/her body to medical science’
	**Spirituality**	3.1 and 3.2	▪ Changes related to these care goals:
			‘Spirituality’ replaced by ‘Religious, spiritual, and cultural needs’
			More space for the nurse to report on these needs
			Anointing of the sick is added
	**Medication**	4.1	▪ Added care goal:
			‘Current medications are assessed and non-essential medications are discontinued’
		4.2	▪ Addition to care goal:
			The anticipatory prescribing of medication for the symptom ‘anxiety’ is added
		4.3	▪ Reworded care goal:
			‘Equipment is available for the patient to support a continuous subcutaneous infusion (CSCI) of medication where required’ replaced by ‘If no intravenous or subcutaneous infusion already in place, the need for a subcutaneous infusion is reviewed’
	**Explanation of the plan of care**	9.5	▪ Added care goal:
			‘The patient’s care providers involved in the hospital and in home care are notified that the patient is dying’
2		c	▪ Reworded care goal:
			‘The patient does not have respiratory tract secretions’ replaced by ‘The patient does not experience discomfort of the respiratory tract secretions’
		k	▪ Reworded care goal:
			‘The patient receives fluids to support their individual needs’ replaced by ‘The need for hydration is reviewed by the multidisciplinary team’
		p	▪ Care goals p and q from the UK are combined:
			‘The psychological well-being of the family carer and the patient are maintained’
		q	▪ Added care goal:
			‘Care givers are able to provide the necessary care’
		r	▪ Added care goal:
			‘The patient/family carer is informed about the patient’s condition’
		s	▪ Added care goal:
			‘The patient/family carer in informed about any change in the plan of care’
2. Supportive documentation
  A goal data dictionary and information leaflets for health care staff and for family carers were developed.
  The goal data dictionary was based on the Dutch version and slightly adapted in compliance with the content of the new Care Guide.
  Four information leaflets were developed based on the Dutch versions: a leaflet for health care professionals about the Care Guide and three leaflets for family carers about the entering of the dying phase, grief, and bereavement after death and facilities available on the acute geriatric ward.
3. Implementation guide
  To help health care staff implementing the care programme for the last days of life in acute geriatric hospital wards, an implementation guide was developed. Table 3 shows the different components of our implementation guide and what they are based on.

**Table 3:** Overview of the components within the implementation guide for the acute geriatric ward

**Components**	Source*
**Component 1-Establishing the implementation project and preparing the environment**	
❖ Informing the health care staff caring for older hospitalized patients about the implementation project and the importance of change in care during the last days of life	1
❖ Executive endorsement: acquiring management approval for the trainings and audits	
❖ Involvement of specialist palliative care services is recommended: at least one member of the Palliative Support Team of the hospital is member of the steering group	1
	1
❖ Facilitators: a nurse and a physician of the geriatric ward	1, 2
❖ Formation of steering group: at least four people of the geriatric ward (facilitators included)	1
❖ Intensive 2-day training of facilitators	1, 2
**Component 2-Preparing the documentation**	
1. Development of an information leaflet for family carers about the facilities in the geriatric ward	1
**Component 3-Baseline review**	
1. Analyzing end-of-life care data of deceased older hospitalized patients using the patients’ medical files	1, 2
2. Feedback of the results to the staff and focusing on improvement within the geriatric ward	
**Component 4-Training health care staff caring for older hospitalized patients**	
1. Facilitators and specialist palliative care colleagues train health care staff with the aid of a training package (i.e. hand-outs with information about the Care Guide, a copy of the Care Guide, a casus to discuss in group etc.)	1, 2
2. Aim training	1, 2
○ To understand and work with the Care Guide	
**Component 5-Care Guide use and intensive support**	
1. Care Guide use after sufficient training and education	1, 2
2. Intensive support and supervision by the steering group through repeated coaching, telephone, and direct guidance, discussion of clinical cases, and clinical audits	1, 2
Component 6-Semi-intensive support	
1. Semi-intensive support and supervision by the steering group through repeated coaching, telephone, and direct guidance, discussion of clinical cases, and clinical audits	1, 2
**Component 7-Evaluation**	
1. To organize a qualitative evaluation of the implementation: evaluating and discussing the performance and progress of each of the previous components	1, 2
2. The qualitative evaluation acknowledges areas where further support, education, or training is need	1
**Component 8-Consolidation**	
1. To adopt a strategy to maintain/improve the implementation and sustainability of the Care Guide	1
2. Support and supervision by the steering group through repeated coaching, telephone, and direct guidance, discussion of clinical cases, and clinical audits	1, 2
**Component 9-Ongoing education, training, and support**	
1. Keeping up to date with developments in end-of-life care and a continuing education and evaluation within the hospital ward	

Source*

1: based on the results of the review of the LCP programmes from the UK, Italy, and the Netherlands.

2: based on the results of the literature review on key factors affecting a successful LCP implementation.
  The implementation guide takes into account most of the components identified in the reviewed LCP programmes and the key factors for a successful LCP implementation.

## Discussion

This article describes the development of a care programme for the last days of life for the older hospital population consisting of a Care Guide for the Last Days of Life, supportive documentation, and an implementation guide to help health care staff in implementing the Care Guide on the acute geriatric ward and to standardize the implementation process across different wards.

An important strength of our study is that it uses the MRC Framework for the development of a complex intervention as a conceptual and methodological basis. This framework, following a five phase iterative approach from pre-clinical phase to large-scale implementation, has proved to be valuable in guiding the development, modelling, and evaluation of complex interventions [26].

To our knowledge, the developed care programme is the first programme that aims to improve care in the last days of life for the older hospital population.

Some limitations have to be acknowledged. Firstly, only three existing LCP programmes were reviewed. As the LCP has been implemented in more than 20 countries, reviewing LCP programmes from other countries could possibly have provided us with more information for the development of our care programme. However, the components of the three reviewed LCP programmes were similar which suggests that a more extensive review would not necessarily have had any added value.

Secondly, we did not perform a systematic review concerning the key factors for a successful LCP implementation. However, the key factors identified in our study largely correspond with key factors identified in a more recently published systematic review [50]. Only one additional contextual factor was mentioned in the review. It was found that a major cultural shift is needed to change the perception from dying as a failure of medical care into dying as a time of life when care takes priority over cure [50]. Also findings from a recent Dutch qualitative study evaluating barriers and facilitators to LCP implementation confirm the key factors identified in our study [51].

Older patients, especially those dying in hospital are a specifically vulnerable patient group for which end-of-life care can be significantly improved [13, 14]. Despite advances in palliative care, hospitalized older patients do not have access to palliative care services in the proportions that might be expected [52] and do not often die a peaceful death, due to prolonged aggressive life-sustaining treatments [53-55]. There is considerable evidence of underassessment and under treatment of symptoms such as pain in hospitals [52]. Moreover, studies have shown that hospitalized elderly people are less likely to receive appropriate pain control and more often receive burdensome interventions at the end of life than do their younger counterparts [6, 10, 56, 57]. However, to our knowledge, no initiative has yet been developed or implemented in order to improve end-of-life care in acute geriatric wards in Flanders.

The care programme for the last days of life, developed for the older hospital population, can be considered as being different from the original LCP programme in several ways. It is specifically adapted to the older hospital population and setting although only small changes were deemed necessary by the reviewers. In the care guide, more attention is paid to specific care goals such as those related to communication, medication and existentialism/spirituality/religiosity and the content of the implementation guide was adapted in such a way that an acute geriatric ward would be better able to implement the Care Guide within its own setting.

The care programme for the last days of life also took into account most of the concerns regarding the use of the LCP raised in the UK. An independent review recently highlighted and confirmed these concerns and subsequently recommended phasing out the LCP in the UK by July 2014 [24]. First, the terminology was changed from ‘Pathway’ to ‘Care Guide’. This might prevent misconceptions about the LCP, such as those among health care staff and family carers and patients in the UK who have perceived ‘pathway’ as a ‘route to death’ [34, 35, 38]. The term ‘Care Guide’ suggests that the document is supposed to guide the health care staff in making individualized choices in caring for dying patients, without being a protocol that has to be followed. This change in terminology was later also recommended in the Neuberger review [24]. Secondly, the importance of a thorough and correct implementation of the Care Guide, underpinned by education, and training, is stressed in our implementation guide. Therefore it incorporates nine components to be performed and includes a detailed and elaborate training package to help health care staff in educating and supporting their colleagues in using the Care Guide in a correct and compassionate way. This counters the identified problem of poor or improper implementation of the LCP in the UK leading to cases of inadequate end-of-life care in the hospital setting [34, 35, 38]. The Neuberger review indeed also confirmed that when the LCP is correctly applied it helps patients to have a dignified and pain-free death. Sufficient training and education should prevent staff from using the Care Guide as a ‘tick box’ exercise instead of as a guidance tool to assist them in decision-making in accordance with a patient’s individual needs [24].

However, not all identified components, and key factors could be incorporated into our implemention guide. First, since there is no central coordinating LCP office in Belgium, the project registration with, and the support by a central LCP office, which is part of all LCP programmes and an important key factor for a successful LCP implementation, could not be included in the implementation guide. Secondly, training of health care staff, is included in the implementation guide but is limited to understanding and working with the Care Guide. Education related to providing good end-of-life care such as symptom control and communication is not part of the training. Nevertheless, the steering group-responsible for the coordination of implementation and consisting of at least one physician, two nurses, and a member of the Palliative Support Team (PST)-is recommended to identify and tackle problems or difficulties in the provision of good end-of-life care during the whole implementation process and can organize additional training if deemed necessary.

Finally, funding for efforts such as facilitation of the implementation process was not available and was thus not included in the implementation guide.

Although the LCP is an evidence-based framework founded on high quality medical practice in palliative care, the Neuberger review underlined the lack of research on the effectiveness of the LCP and on how factors can result in better or worse implementation [24]. We will therefore perform a phase 2 study to evaluate the feasibility of the implementation process and to identify potential problems and difficulties in implementation and use of the care programme in the acute geriatric hospital wards. Based on the results of this phase 2 study we will be able to refine our preliminary care programme. Having developed and modelled this specific care programme it will be important to evaluate its effectiveness thoroughly.

## Conclusions

Performing a phase 0–1 study according to the MRC Framework helped us to develop a care programme for the last days of life for older patients dying in the acute geriatric hospital wards. With the relevant background information we were able to develop a new care programme which takes into account the concerns regarding the LCP in the UK.

## Abbreviations

LCP: Liverpool Care Pathway
MRC: Medical Research Council
PST: Palliative Support Team
